# Aggregation of 53BP1 and XRCC1 in cancer stem cells and non-stem cancer cells post-targeted proton irradiation

**DOI:** 10.1093/jrr/rrt156

**Published:** 2014-03

**Authors:** Gen Yang, Teruaki Konishi, Alisa Kobayashi, Takeshi Maeda, Yukio Uchihori, Tom K. Hei, Yugang Wang

**Affiliations:** 1 State Key Laboratory of Nuclear Physics and Technology, Peking University, Beijing 100871, PR China; 2 Space Radiation Research Unit, International Open Laboratory, National Institute of Radiological Sciences, 4-9-1 Inage-ku, Chiba-shi 263-8555, Japan; 3 Center for Radiological Research, Columbia University, VC11-205, 630 West 168th Street, New York, NY 10032, USA

**Keywords:** cancer stem cells, 53BP1, SPICE microbeam facility, proton irradiation

## Abstract

Tumor cells often exist in different phenotypes with distinct properties. Ever-increasing evidence strongly supported the existence of the cancer stem cells (CSCs) and non-stem cancer cells (NSCCs) in various tumors. It is of fundamental importance to understand the properties of the phenotypes under radiation and chemical treatments, for CSCs are regarded as the source of tumor dormancy as well as recurrence after apparently successful debulking of human solid tumors by various forms of therapy. To understand the DNA damages and repairs in CSCs/NSCCs post-radiation, currently sorted CSCs/NSCCs were irradiated with microbeam irradiation system, SPICE of NIRS [ 1]. The results showed that the aggregation of 53BP1 and XRCC1 are in a dose-dependent manner from 20 to 200 protons per nucleus targeted with proton microbeam of 2 µm in diameter. In addition, compared with NSCCs, there is lower average-related fluorescence unit in 53BP1 foci induced by proton radiation, indicating CSCs might be more radio-resistant than that of NSCCs. Importantly, significant higher diffusion rate of the fluorescence also observed in CSCs than that of NSCCs, indicating that CSCs may have higher repair efficiency than that of NSCCs post-proton irradiation. In addition, the radiation-induced bystander effects in CSCs and NSCCs were also studied via the aggregation of 53BP1.
